# Tissue-engineered trachea from a 3D-printed scaffold enhances whole-segment tracheal repair

**DOI:** 10.1038/s41598-017-05518-3

**Published:** 2017-07-12

**Authors:** Manchen Gao, Hengyi Zhang, Wei Dong, Jie Bai, Botao Gao, Dekai Xia, Bei Feng, Maolin Chen, Xiaomin He, Meng Yin, Zhiwei Xu, Nevin Witman, Wei Fu, Jinghao Zheng

**Affiliations:** 1 Department of Cardiothoracic Surgery, Shanghai Children’s Medical Center, School of Medicine, Shanghai Jiao Tong University, 1678 Dong Fang Road, Shanghai, 200127 China; 2 Institute of Pediatric Translational Medicine, Shanghai Children’s Medical Center, School of Medicine, Shanghai Jiao Tong University, 1678 Dong Fang Road, Shanghai, 200127 China; 3 Department of Cell and Molecular Biology, Karolinska Institute, Stockholm, S-171 77 Sweden

## Abstract

Long segmental repair of trachea stenosis is an intractable condition in the clinic. The reconstruction of an artificial substitute by tissue engineering is a promising approach to solve this unmet clinical need. 3D printing technology provides an infinite possibility for engineering a trachea. Here, we 3D printed a biodegradable reticular polycaprolactone (PCL) scaffold with similar morphology to the whole segment of rabbits’ native trachea. The 3D-printed scaffold was suspended in culture with chondrocytes for 2 (Group I) or 4 (Group II) weeks, respectively. This *in vitro* suspension produced a more successful reconstruction of a tissue-engineered trachea (TET), which enhanced the overall support function of the replaced tracheal segment. After implantation of the chondrocyte-treated scaffold into the subcutaneous tissue of nude mice, the TET presented properties of mature cartilage tissue. To further evaluate the feasibility of repairing whole segment tracheal defects, replacement surgery of rabbits’ native trachea by TET was performed. Following postoperative care, mean survival time in Group I was 14.38 ± 5.42 days, and in Group II was 22.58 ± 16.10 days, with the longest survival time being 10 weeks in Group II. In conclusion, we demonstrate the feasibility of repairing whole segment tracheal defects with 3D printed TET.

## Introduction

Trachea stenosis is a rare but life-threatening condition in patients. In adults, it is usually caused by prolonged endotracheal intubation, tracheostomy, trauma, trachea cancer and inflammation^1^. In children and apart from the aforementioned causes listed above, trachea stenosis may also be a congenital abnormality. For several decades, many surgeons have made great effort in seeking novel treatments for this disease. Tracheal resection and re-anastomosis existed as a clinical solution as early as the late nineteenth century, but it was contraindicated for stenotic segments longer than 2 cm in children and 5 cm in adults due to the risk of excess tension^2^. Recently, slide tracheoplasty has improved outcomes significantly for preciously inoperable patients^3, 4^. However, performing such a procedure comes at a sacrifice of tracheal length for tracheal inner diameter. In our center alone we have nearly ten-years’ experience with the surgical management of congenital tracheal stenosis; still we find current surgical strategies have major limitations regarding the location and length of trachea stenosis, as well as postoperative problems with tracheomalacia and granulation formation^3^. One novel solution to overcoming these obstacles is the use of artificial substitutes to replace long-segment narrowed trachea.

Many studies have turned to grafting technologies to overcome the unmet clinical needs facing tracheal repair. However, allografts and autografts are limited due to a severe lack of healthy donors and a heightened risk of immune-rejection^5, 6^, and synthetic materials are limited in biocompatibility and granulation formation^7, 8^. In contrast, because of the proper autologous availability, Tissue-Engineered Trachea (TET) seems to be an ideal solution for trachea replacement^9^. By choosing suitable materials for scaffolds and seeding these scaffolds with homogenous cells, TET can emulate similar biological structures and functions to that of native trachea. Furthermore, with the progress of 3D printing techniques, it is possible to design an individualized tracheal model that is suitable for the host in morphology, as well as properly support force to maintain the shape of the TET^10, 11^.

In recent years, several authors have attempted to make trachea scaffolds through the combination of Tissue-Engineering and 3D-Printing techniques^12–16^. However, these studies only aimed to fix the anterior-end defects of tracheas and further studies are needed in order to demonstrate similar technologies could be employed for whole segment tracheal replacement. A recent study successfully reported construction of whole TET with mechanical properties similar to that of the native trachea by a 3D printing scaffold^17^. Still, a major caveat in this study was the complexity of the manufacturing of the TET. Additionally, the study lacks any *in vivo* evidence that the TET could be used in experimental replacement surgery.

In this study, we 3D-printed a scaffold of the whole segment of rabbit trachea, which had similar morphology to that of the native trachea as well as the proper support force to maintain the lumen. A biodegradable material, PCL, was used to build this scaffold due to its appropriate melting point and malleability for 3D printing. We also presented a convenient method to achieve cartilage tissue regeneration and TET reconstruction, and used these TETs to replace the native rabbit trachea, *in vivo*, to demonstrate the feasibility and potential problems associated with these TETs.

## Results

### Printing of PCL scaffold

**Figure 1:**
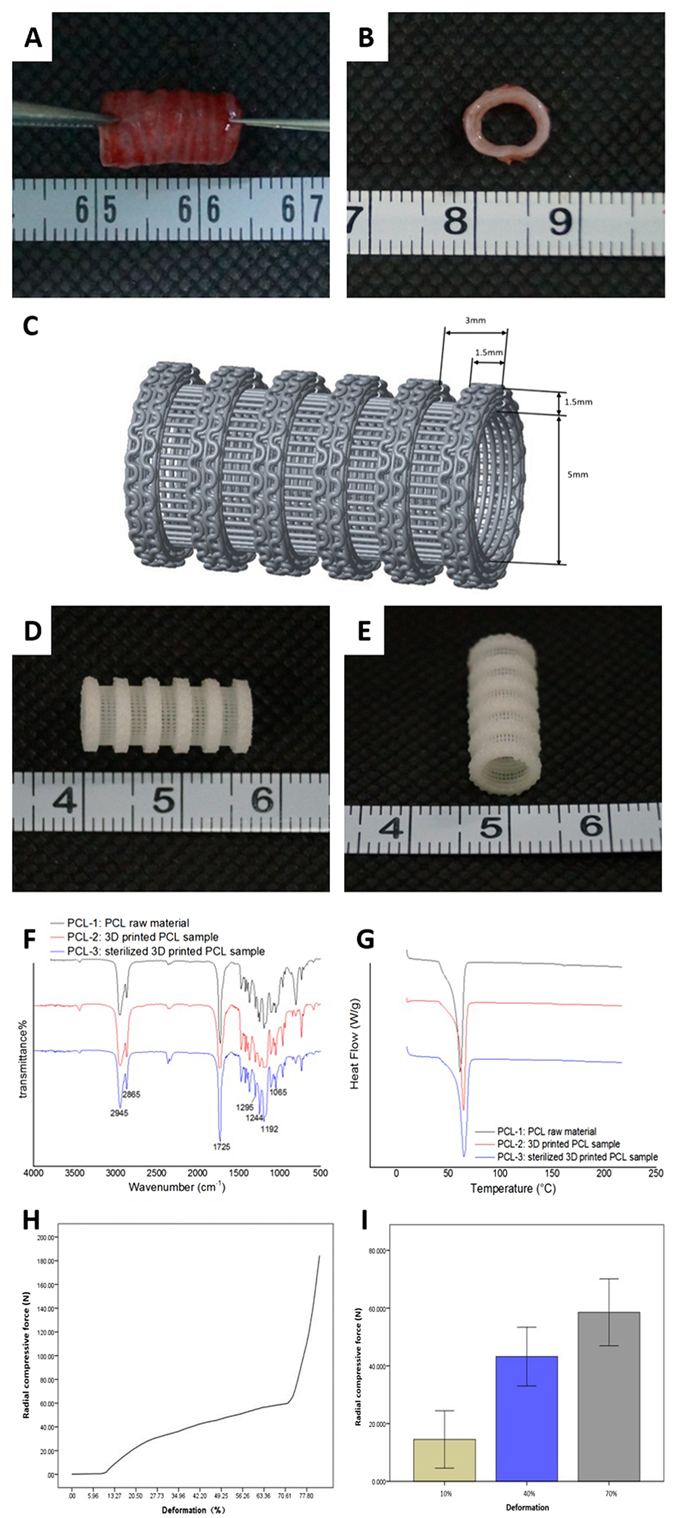
Design of a novel 3D-printed whole trachea from PCL scaffold. (**A** and **B**) Representative photograph of native trachea extracted from a four-month-old Zealand white rabbit. (**C**) Blueprint design of a 3D printed PCL scaffold. (**D** and **E**) Representative photograph of a 3D printed PCL scaffold. (**F**) Representative FT-IR spectra of PCL raw material, 3D-printed PCL samples, and sterilized 3D-printed PCL samples. The characteristic peaks for carbonyl stretching is shown at 1725 cm^−1^ and spectra present at 1295 cm^−1^ reveal the backbone C-C and C-O stretching modes both of which revealed no major differences between the three groups. The asymmetric and symmetric C-O-C stretching can be confirmed by the peaks present at 1244 cm^−1^ and 1192 cm^−1^, respectively, and peaks at approximately 1065 cm^−1^ are associated with the COH stretching, where again the pattern is maintained across all three groups. (**G**) Differential Scanning Calorimetry (DSC). Results indicate same amount of heat is required to raise the temperature of the PCL raw material, 3D-printed PCL samples, and sterilized 3D-printed PCL. (**H**) Representative radial compressive force-deformation curve of scaffold. (**I**) Average radial compressive force highlighting 10%, 40% and 70% deformation (n = 3).

We carefully designed a PCL scaffold that closely resembled the dimensions to that of the native trachea of a 4-months old New Zealand white rabbit (Fig. 1A and B). The longitudinal length measured 1.65 cm and consisted of 6 convolutions. The thickness of each convolution was 1.5 mm and the distance between two convolutions was 1.5 mm. The luminal diameter measured 5 mm which is very similar to that of the normal average diameter of a rabbit’s native trachea^18^. The inner layer of the scaffold consists of vertical and horizontal PCL cross bars. The PCL cross bars have a diameter of 0.12 mm, which simultaneously acts as a support system to strengthen the scaffold while providing enough space to coat those areas with chondrocytes. The grids of the PCL bars are 0.24 * 0.12 mm. Figure 1D and E show a representative photograph of a 3D printed PCL scaffold.

In order to ascertain if any structural changes incurred to the chemistry of the PCL during the 3D-printing process of the TET, we carried out Fourier Transform Infrared Spectroscopy (FT-IR) analysis. We analyzed absorption spectra at several wavelengths, which correspond to different carbon compounds found in the PCL material. Analysis revealed absorption spectra patterns were not altered between the three groups of PCL raw material, 3D-printed PCL scaffolds, and sterilized 3D-printed samples (Fig. 1F).

Additionally, to verify the thermoanalytical properties were also similar between the raw PCL material, 3D-printed PCL scaffolds and the sterilized 3D-printed PCL, we performed Differential Scanning Calorimetry (DSC) assays. We found sharp endothermic peaks at around 60 °C in all three groups, which correlates to the melting points of the PCL materials (Fig. 1G). During a 3D-printing process of PCL, chamber temperatures are set to 90 °C and orifice temperatures are set to 110 °C. According to the DSC results shown, no obvious thermal decomposition was observed between all three groups in this temperature range. Therefore, we conclude that our current 3D-printing method does not cause changes in the chemical structure of PCL. Additionally, no change in the PCL crystalline structure was observed before and after the sterilization process (Fig. 1F and G). These results show that the 3D-printing method we employ is a reliable and highly efficient way to manufacture tissue engineering scaffolds.

Next, to get a practical perspective on the mechanical properties of the 3D-printed scaffold we performed radial compressive force-deformation analysis (Fig. 1H and I). According to the curve shown in Figure H, small forces of 20 N cause slight deformation, levels equivalent to 10–20% deformity. However, with a radial compressive force of 60 N, we observe 70% deformation at which point we observed the inner lumen of the scaffold becoming crushed. The radial compressive force at 14.52 ± 9.95 N, 43.20 ± 10.17 N and 58.55 ± 11.60 N correlates with 10%, 40% and 70% deformation respectively. We believe these results show that our 3D-printed TET has favorable support function for long-term *in vivo* engraftment.

### Reconstructed TET *in vitro*

**Figure 2:**
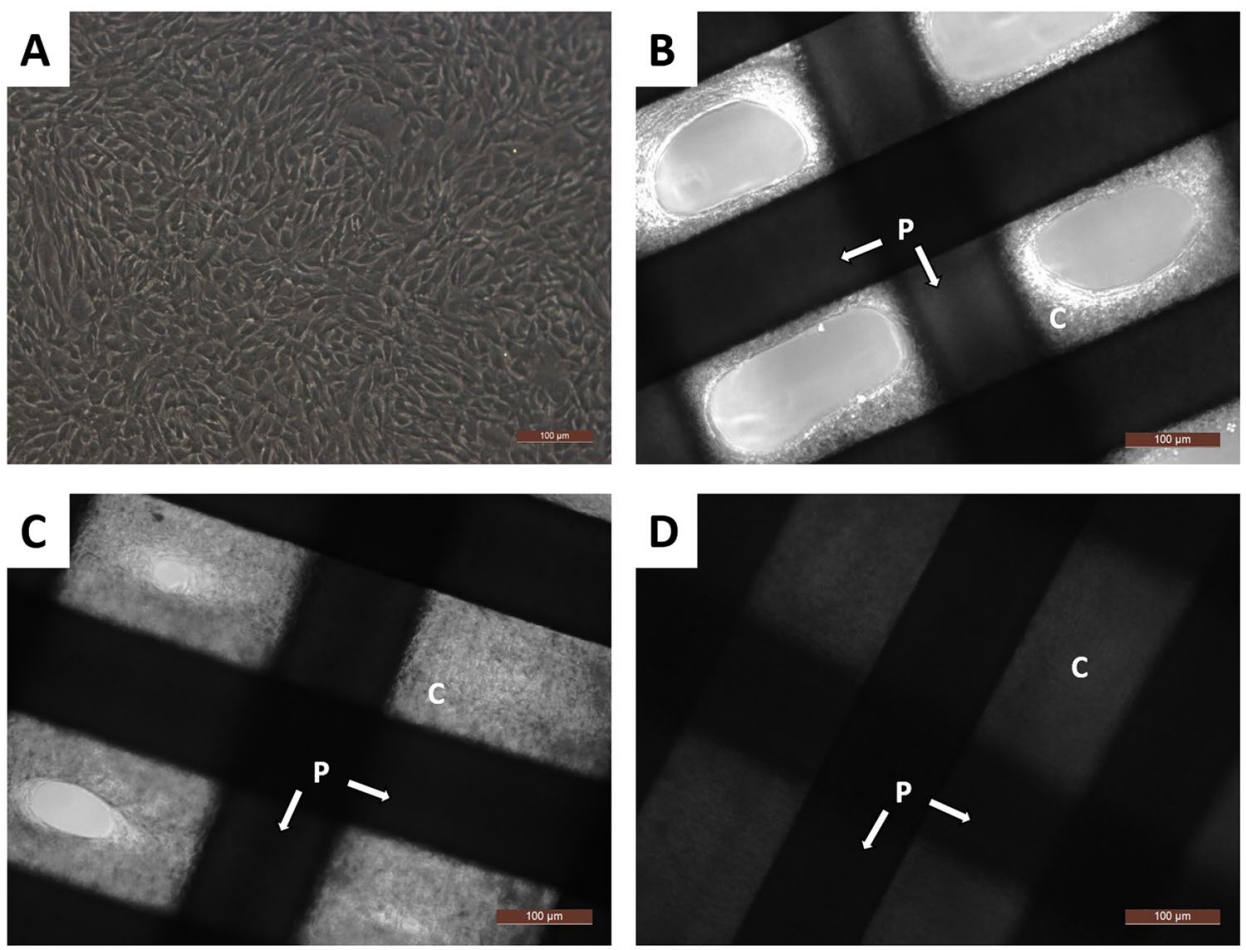
Observation of cell-adhesion to the PCL scaffold with light microscopy. (**A**) Morphology of chondrocytes on second passage using a light microscope. (**B**) 3 Days, (**C**) 7 Days, (D) 14 Days post incubation. Note the decrease of light penetrance on the PCL bars (P) in Fig. 2C and D compared to Fig. 2B due to scaffold incubation with chondrocytes for 1 and 2 weeks, respectively. Scale bar: 100 μm. (P: PCL bars. C: chondrocytes and extracellular matrix).

In order to produce a scaffold saturated in cartilaginous tissue, we cultured our 3D-printed scaffold *in vitro* with seeded chondrocytes. Favorable adhesion of the chondrocytes to the PCL scaffold was observed by microscopy (Fig. 2A–D). After gently shaking the chondrocyte-scaffold culture mix for 3 days, chondrocytes gathered around the PCL bars leaving unfilled spaces between the convolutions (Fig. 2B). However, by day 7 migrating chondrocytes began to immerse the PCL cross bars (Fig. 2C), and by day 14 the entire scaffold was filled with chondrocytes and secreted matrix components. This is denoted by the large decrease in the transmittance of light throughout the cross bar segments of the scaffold (Fig. 2D). Together, these data indicate the chondrocytes maintain good proliferation capacity and material affinity on the scaffold.

**Figure 3:**
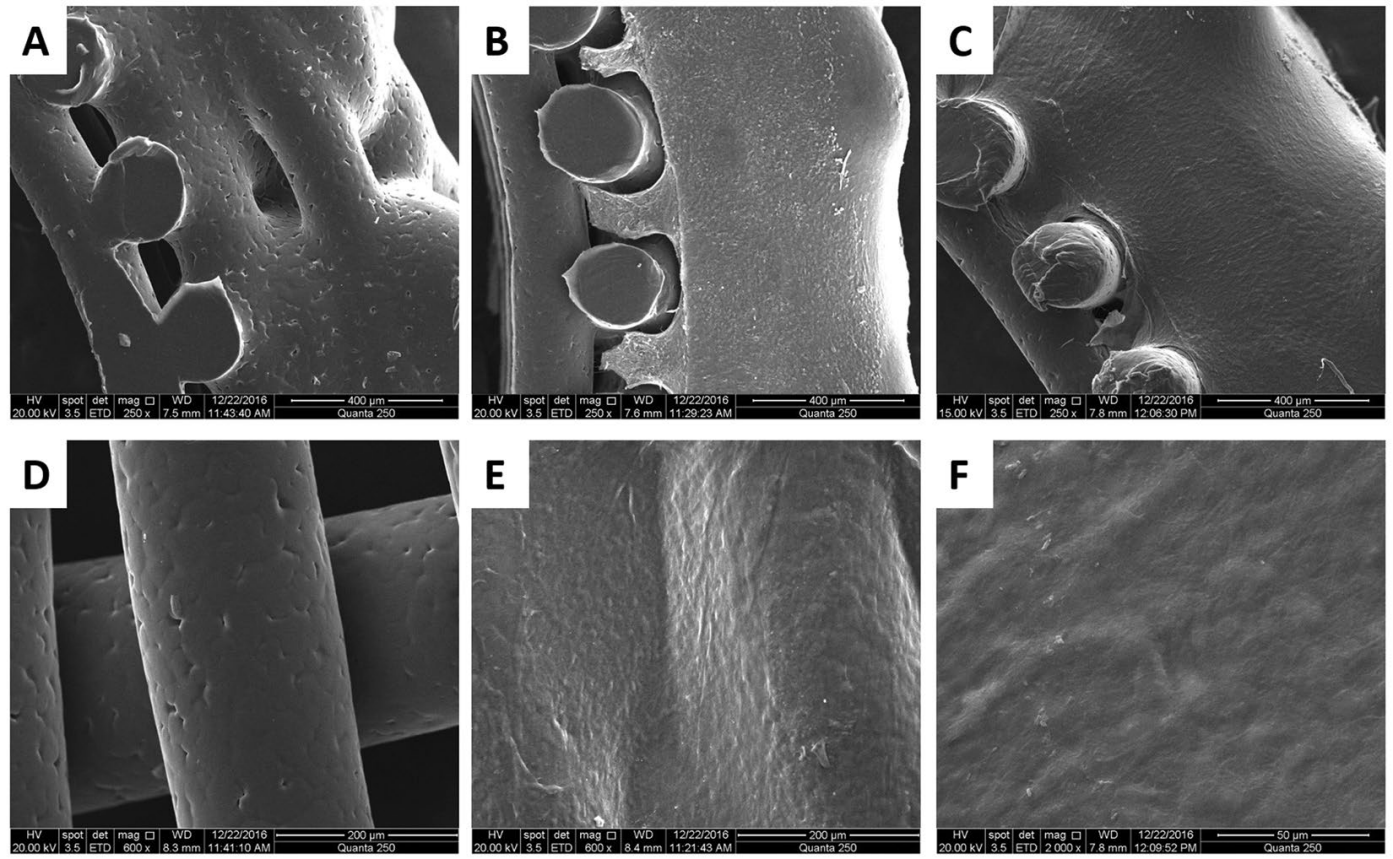
Observation of TET by SEM. Transverse sections of the convolutions of (**A**) Blank scaffold, (**B**) 2-weeks cultured TET, (**C**) 4-weeks cultured TET. (**D**) Surface of bare-PCL bars. (**E**) Surface of 4-weeks TET, note PCL bars presented with an ECM-like appearance covering the surface. (**F**) Enlarged image of the surface of the ECM-like tissue on the TET. Scale bars: (**A**–**C**) 400 μm, (**D** and **E**) 200 μm, (**F**) 50 μm.

**Figure 4:**
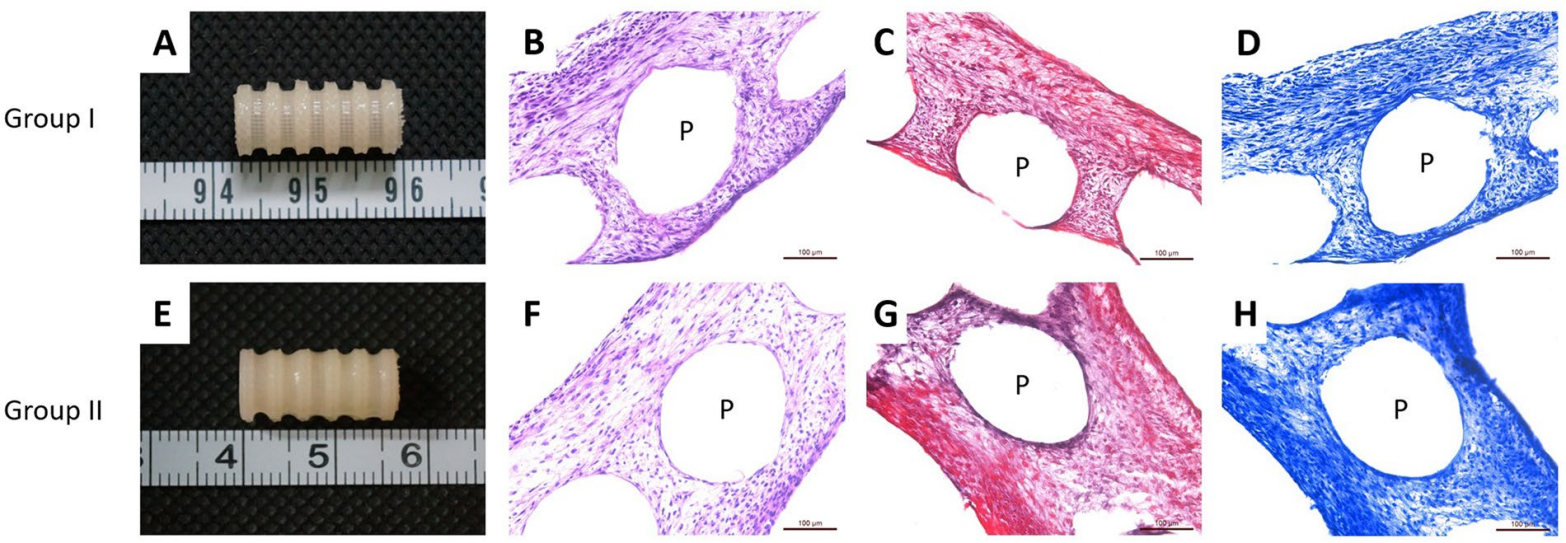
Representative photomacrographs and histological stainings of tissue-engineered tracheas after reconstruction *in vitro*. (**A**–**D**) Group I, TET *in vitro* cultured with chondrocytes for 2 weeks. (**E**–**H**) Group II, TET *in vitro* cultured with chondrocytes for 4 weeks. (**A**,**E**) Gross view of TET. (**B**,**F**) Histology staining with H&E. (**C**,**G**) Histology staining with Safranin O. (**D**,**H**) Histology staining with Toluidine blue. Scale bars: 100 μm. (P: PCL scaffold bars).

After being cultured for 2 or 4 weeks *in vitro*, the TETs were taken out of the incubator for Scanning Electron Microscopy imaging (SEM), photo-macrograph analysis and histological analysis (Figs 3 and 4). The PCL scaffold showed good cytocompatibility with chondrocytes (Fig. 3). The surface of the scaffold was covered with a thin layer of ECM-like tissue. Both pores in convolutions and grids between PCL bars were fully filled with matrix tissue (Fig. 3B,C,E,F). In the 4-week cultured TETs, the chondrocytes fully engulfed the scaffold and assembled a cell layer with the appearance of a secreted extracellular matrix (Fig. 3F).

Following *in vitro* chondrocyte treatment of the scaffolds for a short incubation period, Group I, or a longer incubation period, Group II, we performed detailed histological analysis on the TETs. In the photo-macrograph images seen in Fig. 4, the Group I TET construct presented a fine layer of ivory-white tissue with a smooth surface (Fig. 4A–D). All grids were covered with a thin translucent tissue-film while the convolutions formed thicker tissue. In Group II, a more mature and dense cartilage-like tissue appeared molded on the TET, leaving the PCL bars difficult to observe (Fig. 4E–H).

Detailed histological analysis of the TET treated scaffolds confirmed that neither group formed mature cartilage tissue (Fig. 4). In Group I, the arrangement of the cells appeared disordered and histological staining with Safranin O and Toluidine blue indicated minimal presence of a cartilage extracellular matrix. However no cartilage lacuna formation was observed. Similar results was also observed in Group II, although cell arrangement was more uniform and the whole tissue seemed more mature, no cartilage lacuna was observed by histological staining.

### Implantation of TET in nude mouse

**Figure 5:**
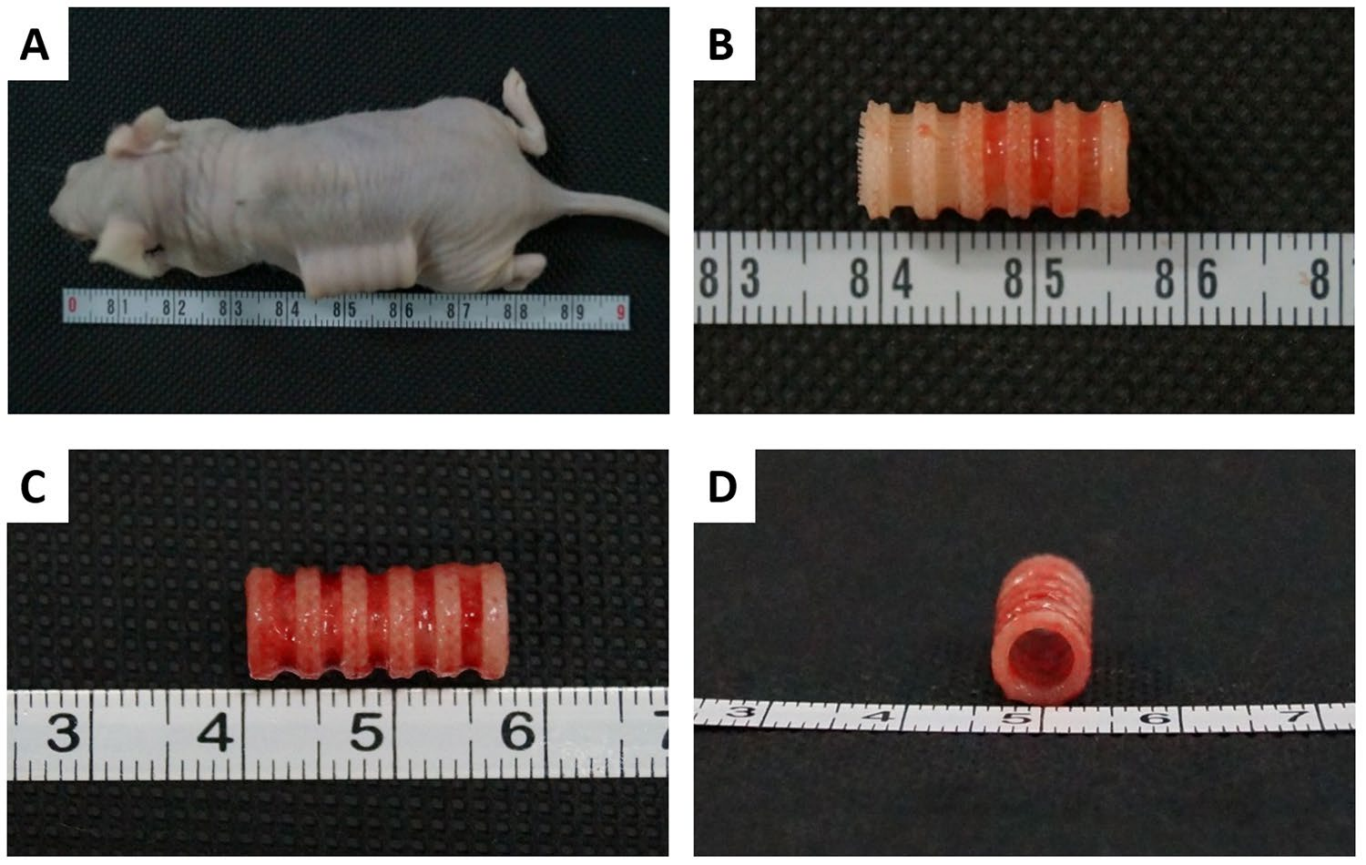
Representative photomacrographs of tissue-engineered trachea post-implantation and immediately after being harvested from nude mice. (**A**) Nude mice with implanted TET. (**B**) Retrieval of Group I implanted TET. (**C**) Retrieval of Group II implanted TET. (**D**) Lateral view of a harvested TET from Group II.

In order to determine if the scaffold was prone to adherence of a mature matrix following incubation with chondrocytes, we decided to implant the TET in the dorsal subcutaneous spaces of nude mice. We harvested the TETs at 6 weeks-post implantation for detailed evaluation (Fig. 5). Both Group I and Group II treated TETs presented mature cartilaginous tissue as depicted by gross photomicrographs (Fig. 5B and C). TETs in both groups formed a more densely covered tissue on the PCL scaffold. The scaffold showed good support and maintained its original shape. Both groups were covered with little connective tissue from the nude mouse, while the lumen remained unobstructed (Fig. 5D).

**Figure 6:**
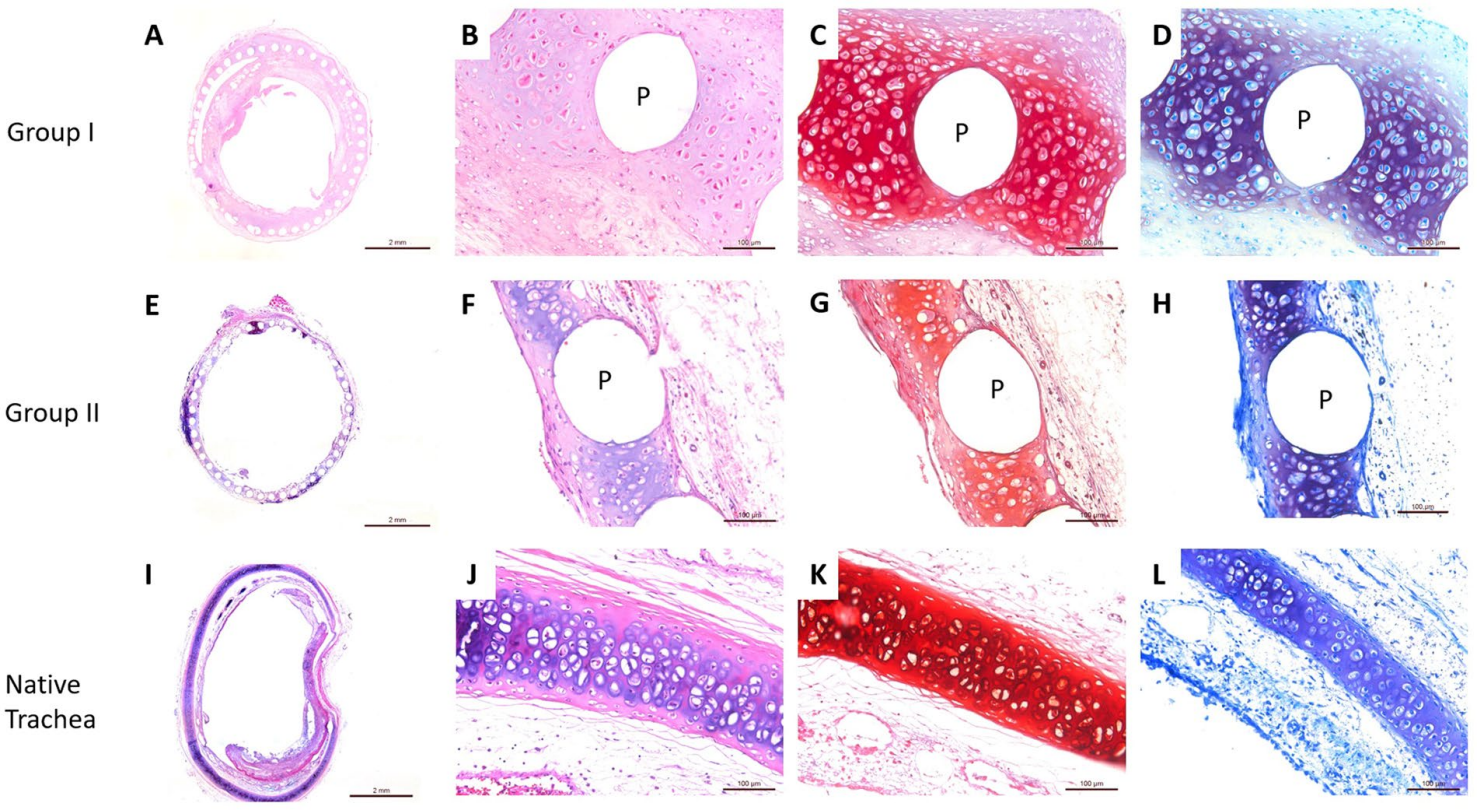
Histological stainings of tissue-engineered trachea *ex vivo* 6 weeks post maturation in nude mice. (**A**–**D**) Group I, *in vitro* cultured TET for 2 weeks. (**E**–**H**) Group II, *in vitro* cultured TET for 4 weeks. (**I**–**L**) Native trachea. (**A**,**B**,**E**,**F**,**I** and **J**) Histological stainings with H&E. (**C**,**G** and **K**) Histological stainings with Safranin O. (**D**,**H** and **L**) Histological stainings with Toluidine blue. Scale bars: (**A**,**E** and **I**) 2 mm, (**B**–**D**,**F**–**H**, and **J**–**L**) 100 μm. (P: PCL scaffold bars).

After the 6 week incubation time in nude mice, the TETs were processed for histological analysis and again compared to that of the native trachea (Fig. 6). The histology revealed that both TET groups had formed typical cartilage tissue similar to native cartilage. Although little connective tissue from the nude mouse can be observed in the inner lumen of the TET, the whole TET maintained unobstructed (Fig. 6A and E). Around the PCL bars, typical cartilage tissue with cartilage lacuna can be seen in H&E staining (Fig. 6B and F). In the histology stainings with Safranin O and Toluidine blue, proteoglycan of cartilage matrix and chondrocytes were clearly revealed (Fig. 6C,D,G and H). Both Group I and Group II had similar performance and the thickness of cartilage tissue in TET is similar to native trachea (Fig. 6I–L).

### Transplantation of TET in a rabbit model

**Figure 7:**
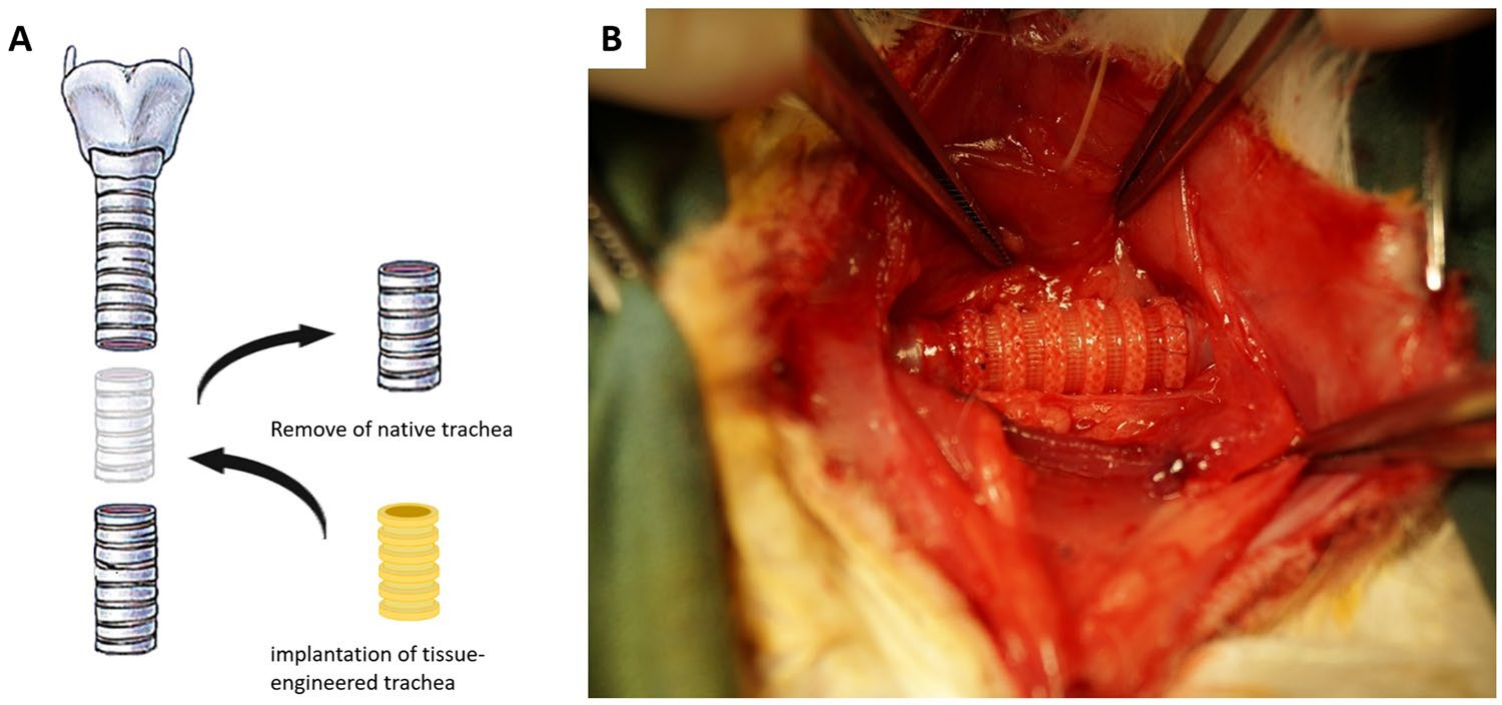
Surgical procedure for the replacement of the native trachea by tissue-engineered trachea. (**A**) Schematic illustration of replacement surgery. (**B**) Tissue-engineered trachea implantation by end-to-end anastomosis.

In order to evaluate the feasibility of repairing whole segment tracheal defects with our *in vitro* treated TET, replacement surgery of rabbit’s native trachea by TET was performed. For all replacement surgery, a segment of native trachea corresponding to the length of our TET was excised; afterwhich the TET was implanted into native trachea by end-to-end anastomosis (Fig. 7A). After reconstructive surgery with the Group I TETs, little exudation around the TET implantation site was observed. With respiration, superficial oscillation of the ECM-like tissue between the PCL bars on the surface of the scaffold could be seen. Following the reconstructive surgery with Group II TETs, no exudation or errhysis was observed following surgery, nor was any superficial movement observed on the surface of the scaffold (Fig. 7B).

One week following the replacement surgery of the native trachea with TET we performed bronchoscopy examination. Animals that underwent bronchoscopy surgery easily succumbed to death due to anesthesia or airway stenosis. However, local inflammation could be detected as well as the formation of granulation tissue in the scaffold segment (data not shown). Due to the poor survival rates associated with bronchoscopy examination, along with the technical hurtle of passing the bronchoscope through the scaffold segment, this test became obsolete on the vast majority of animals undergoing TET surgery.

**Figure 8:**
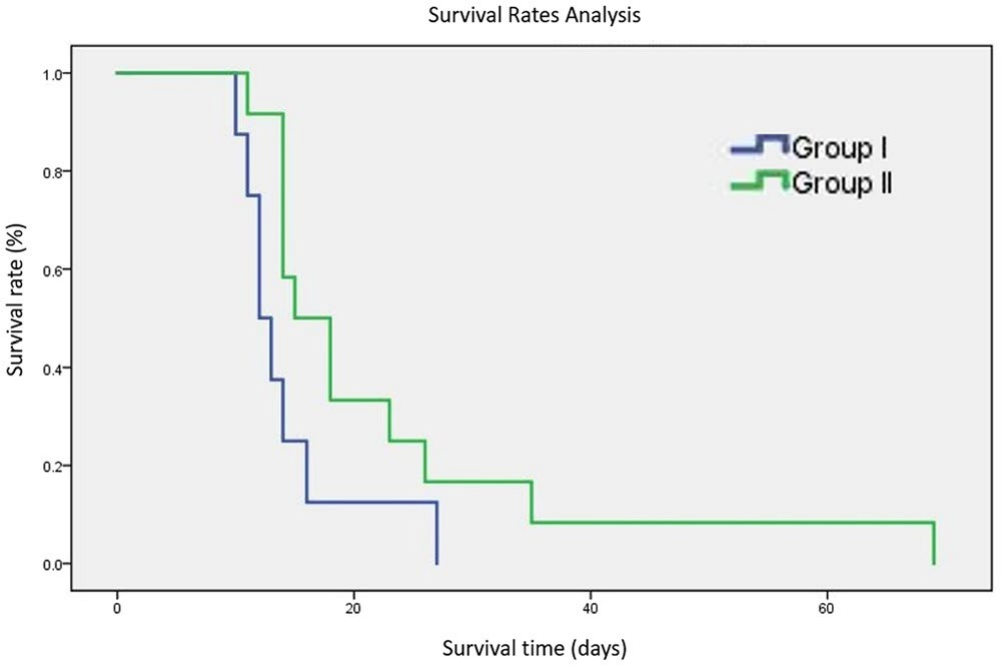
Survival rates analysis after trachea replacement surgery by Kanlan-Meier method and Breslow test. P = 0.034.

Next we sought to compare mean survival rates of animals receiving TET replacement surgery, between the two groups of chondrocyte treated scaffolds. Analyses of the post-operative survival rates are shown below (Fig. 8). Group II treatment groups presented a significantly longer survival time than Group I. In summary, the mean survival time was 14.38 ± 5.42 days in Group I and 22.58 ± 16.10 days in Group II. All the rabbits that received TET replacement surgery died within 10 weeks. The most common cause of death in both groups was granulation formation in TET segments (75%, 15/20 rabbits).

**Figure 9:**
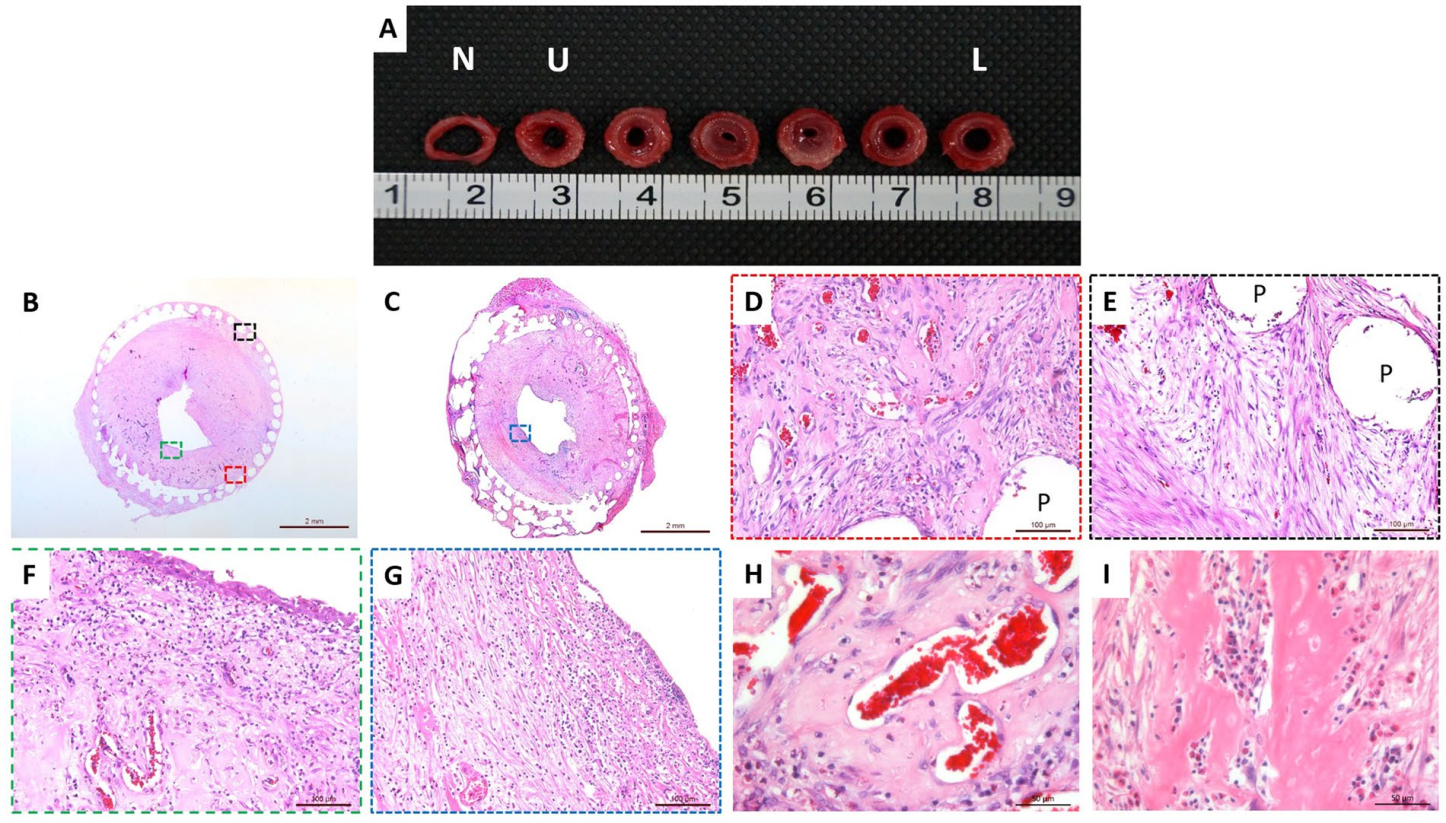
Representative photomicrograph and histological stainings of tissue-engineered trachea after trachea replacement surgery. (**A**) Granulation formation at each convolution throughout a scaffold segment. Note severe stenosis most obvious in middle convolutions (**B**) H&E staining of TET at 3 weeks-post implantation. (**C**) H&E staining of TET at 10 weeks-post implantation. (**D**) Enlarged photomicrograph revealing cartilage-like tissue in the outer layer of the scaffold. (**E**) Enlarged photomicrograph depicting the fibrous-like tissue present in the outer layer of the scaffold. (**F**) Photomicrogaph depicting lack of epithelial infiltration in luminal region at 3 weeks-post transplantation. (**G**) Photomicrograph depicting no evidence of epithelial infiltration at 10 weeks-post transplantation. (**H**,**I**) High resolution imaging depicting vascularization and infiltrating inflammatory cells within the scaffold, respectively. Scalar bars: (**B** and **C**), 2 mm, (**D**–**G**) 100 μm, (**H** and **I**) 50 μm. (N: native trachea; U: upper anastomosis; L: lower anastomosis; P: PCL scaffold bars; Black arrow: residual cartilage tissue).

A detailed autopsy examination was performed at time of death. According to the results from the autopsy, our TETs provided strong functional support to the native operated tracheas. This was made evident by the lack of observed tracheal collapse or malacia at the time of autopsy. The whole segment of TETs displayed some stenosis where the middle sections were the narrowest (Fig. 9A). We observed surrounding tissue adhered to the outside surface of the TETs (Fig. 9B–E). Among the PCL bars, cell arrangement and formation had a tendency to grow from outside to inside, indicating the source of granulation tissue was most likely the connective tissue surrounding the trachea (Fig. 9E). The surface of the lumen of the TET lacked epithelium tissue formation in both groups (Fig. 9F and G).

We did, however, observe deposits of cartilage-like tissue in histological stainings at time of autopsy (Fig. 9D,H and I). We speculate that mature cartilage tissue may have been eroded by surrounding tissue or from a severe inflammatory reaction, as new capillary formation and inflammatory cells were observed surrounding the remaining cartilage-like tissue (Fig. 9H and I).

Furthermore, the autopsies revealed other causes of death, which included pneumonia, infection in scaffold segment and anastomotic stenosis (Table 1). Both infection-related deaths in Group II happened less than 3 weeks after replacement surgery. Scaffold segments had some granulation tissue formation, which caused sputum discharge to be more difficult and increased the risk of infection. Anastomotic stenosis in both groups had one case, and may be attributable to surgery impairment.

**Table 1:** List of death reasons.

Death reasons	Group I	Group II
Granulation formation	7 (87.5%)	8 (66.67%)
Pneumonia	—	2 (16.67%)
Infection in scaffold segment	—	1 (8.33%)
Anastomotic stenosis	1 (12.5%)	1 (8.33%)

## Discussion

Recently, the rise of 3D printing technology provides infinite possibilities for tissue engineering^10, 19^. In this study, we designed and 3D-printed an intact trachea scaffold with similar morphology to that of a native rabbits’ trachea, which presented good cytocompatibility, as well as provided solid functional support in the early stages after replacement operation. This study highlights for the first time, the possibility of repairing whole tracheal defects with TET in an animal model.

In contrast to increasing advances in tissue engineering and grafting technologies, still an unmet clinical need persists in regards to repairing partial tracheal defects using 3D printed scaffolds. To date, several studies have tried to utilize 3D-printing scaffolds, with and without cell-coating pre-treatment steps, in order to repair partial tracheal defects^12–15^. The latter of these two studies reported neocartilage formation at 8–12 weeks following surgical implantation. However, their scaffolds were less than 1 cm in length and only fit partial tracheal defects less than the size of one semicircle of annulus tracheal. Although these studies identified significant breakthroughs of TET, the length and shape of the scaffolds limited the application of the TET.

In order to treat long segments of tracheal stenosis, whole tracheal scaffolds display a more promising artificial substitute. In a current study by Park *et al*., the etteam reported an intact tracheal scaffold with similar morphology to the native trachea and appropriate mechanical behavior similar to native trachea^17^. The reconstructed TET contained a gelatin portion that had been seeded with chondrocytes, allowing the formation of neocartilage tissue following transplantation into the dorsal subcutaneous spaces of nude mice. However, the procedures used here to make such a scaffold are fairly complicated. Furthermore, the most objective criteria of TET replacement surgery are the *in vivo* evaluations of the TET, which the authors did not include in their study.

Here, we presented a novel design for a 3D-printed whole tracheal scaffold by employing a rather simple method. The TET construct presented good cartilaginous properties both *in vitro* and *in vivo*. This is one of the first papers to utilize a 3D-printed TET for rabbit tracheal transplantation and nicely demonstrates the feasibility of repairing whole segment tracheal defects with such a procedure.

Our chondrocyte treated TET scaffold has several advantages worth highlighting: 1) The ployporous structure is conducive to cell adhesion and proliferation, which makes the method of tissue reconstruction simple; 2) The morphology of the scaffold is very similar to that of native trachea and can meet physiological demand. In addition, with 3D-printing technology, it is easy to change the morphology of the scaffold to achieve individualized treatment. 3) As a biodegradable material, PCL has both appropriate support strength and favorable cytocompatibility. The long-term goal would be to achieve new anastomosis through a PCL-decayed, reconstructed cartilage formation enhanced by host epithelization.

One major caveat of our TET design was that our scaffold lacks contractility properties supporting that of a native trachea, and the chondrocyte treated TET needs major modifications in order to tolerate secretion or discharge. Additionally, with 3D-printing technologies, always some design/printing errors may occur, which can render the shape of the scaffold and set it slightly apart to those dimensions of the native trachea.

Irregardless of such pros and cons presented with our TET, repairing whole segment tracheal defects by TET is a complex procedure, especially due to diminishing epithelial tissue within the segment, which can easily cause obstructed secretion, damage of respiratory function and/or infection. The primary postoperative causes of death in our study are immediately relevant with the reasons mentioned above. Granulation formation is a common complication of trachea surgery^2, 20, 21^. This progress, mediated by a wide range of coordinated cellular reactions, has a close relation with infection, inflammation, tissue necrosis and immunological rejection^22, 23^. In our opinion, the stenosis seen in the scaffold segment and granulation formation after transplantation surgery is related to the lack of any protective epithelial layer along with an accompanying inflammatory reaction.

In this study, we report the successful formation of cartilage tissue in the dorsal subcutaneous spaces of nude mice, but such a response failed in the rabbits’ airway. The nude mouse had an inhibited immune system, and subcutaneous space is an aseptic condition. In contrast, in the rabbit, the environmental conditions in the airway are not of a sterile condition, bacterial colonization from the respiratory tract may cause local inflammation, which is harmful for the maturation of cartilage tissue.

Another common complication of tracheal surgery is tracheomalacia, which was not observed in our experiment, indicating a favorable mechanical support of our TET. The mechanical properties of the scaffold is inextricably linked to the strength and diameter of material. Increasing the thickness of the diameter of the scaffold often equates to an increased strength of the scaffold, both of which prolongs degradation time. Here, due to post-transplantation survival rates not exceeding 10 weeks, it is difficult to ascertain the long-term functional duration of our TET. In particular, more information regarding how degradation will alter the reparative processes needs further evaluation. Additional experiments are also needed to ensure scaffold diameters are functionally optimized.

Lastly, in order to maintain function and improve long-term survival and grafting rates, vascularization and epithelization are two important aspects when dealing with TET transplants^12, 23–29^. Angiogenesis and neovascularization provides nutritional support to the implanted TET and have previously been shown to be key factors influencing ingrowth of epithelium^21, 24^. Epithelization provides critical functions such as the prevention of bacteria, as well as ciliary beating which helps enable discharge of secretion. In order to induce vascularization and epithelization which could aid in the long-term survival of TET transplants, several approaches exist, including pre-vascularization *in vivo* ^21, 25^, pretreating the scaffold or cell-scaffold interface with growth factors (such as VEGF)^22, 23^, and/or using human turbinate mesenchymal stromal cell sheets as an inner surface layer in TET for epithelial regeneration^13^.

Another way to reduce inflammation and granulation formulation following transplantation of TET is through inhibiting the immune system. This is easily attainable by using dexamethasone or IL-1 receptor antagonists^22^. However, to perform such studies could enhance a risk of serious infection, requiring more strict conditions for animal housing and postoperative care. At least according to our results, the 4-weeks cultured TET group presented a longer survival time than the 2-weeks cultured TET. Prolonged culture times or culturing scaffolds with more cells may be an additionally helpful way to prolong the maturation of the transplanted TET.

At present, the concept of utilizing a functionally sustained TET for intrathoracic tracheal replacement surgery shows much promise. With the help of 3D-printing, we establish a simple solution for the adhesion of cells on a 3D-printed scaffold and proved the feasibility of cartilage tissue formation. Although there are some experimental setbacks in regards to the animal replacement experiments, we envision the ideas and methods of the scaffold design and tissue reconstruction presented in this study can be a meaningful early-stage determinant, and help to inspire more practicable approaches to repair long-segment trachea stenosis. In order to move forward with clinical application, information within the parameters of morphology, function and degree of comfort are severely required.

## Conclusion

With the combination of 3D-printing and tissue engineering, we 3D-printed an intact tracheal scaffold with biodegradable material and successfully reconstructed TET with a chondrocyte suspension. The TET presented good cartilaginous properties both *in vitro* and *in vivo*. Such technology could one day be considered for tracheal replacement therapies, as we displayed evidence that the TETs can endure long-term implantations, up to 10 weeks survival time *in vivo*. In conclusion we demonstrate the feasibility of repairing whole segment tracheal defects with the use of 3D printed TET.

## Methods

### Experimental animals

A Total of twenty-one New Zealand white rabbits (4-months old) were purchased from Shanghai Chedun Experimental Animal Raising Farm (Shanghai, China). Four male nude mice (7-weeks old) were purchased from Shanghai Slaccas Experimental Animal Ltd. (Shanghai, China). All experimental protocols were complied with the relevant guidelines and regulations and approved by the Animal Care and Experiment Committee of Shanghai Jiao Tong University School of Medicine.

### PCL scaffold design and 3D printing

The 3D printing PCL scaffold was designed exactly as native trachea of a 4-months old New Zealand white rabbit. A widely used biodegradable material, PCL, was used to print the scaffold. All scaffolds were 3D printed by Beijing Advanced Medical Technologies Ltd. Inc. (Beijing, China). Rapid Stent Fabrication System was used as a preparation system of the scaffold.

To further detect whether there are some changes of chemical/material properties, PCL raw material, 3D printed PCL sample and irradiation sterilized 3D printed PCL sample were tested by Fourier Transform Infrared Spectroscopy (FT-IR) and Differential Scanning Calorimetry (DSC).

### Radial compressive force measurement

Three scaffolds were compressed on a computer stretch and compression tester (HY-940FS, Hengyu, Shanghai, China). The presser foot controlled compression of the scaffold with the speed of 2 mm/min. The radial compressive force and the percentage of deformation was recorded for the Radial compressive force-deformation curve. All compressing tests were conducted under standard environment conditions (20 ± 1 °C, RH 65 ± 2%).

### Isolation and culture of chondrocytes

The rabbit chondrocytes were isolated as previously described^30^. Briefly, primary cartilage tissue with a size of about 2.0 * 3.0 cm was obtained from auricle of rabbit and separated under sterile conditions. The cartilage slice was washed with phosphate buffered solution (PBS; Hyclone, Logan, UT, USA) and digested with 0.25% trypsin plus 0.02% Ethylene Diamine Tetraacetie Acid (EDTA) (Gibco, Waltham, MA, USA) at 37 °C for 30 minutes, minced into approximately 1 mm^3^ pieces, and then digested with 0.1% collagenase NB4 (SERVA, Heidelberg, Germany) in serum-free Dulbecco’s Modified Eagle’s Medium (DMEM; Hyclone, Logan, UT, USA) at 37 °C for about 12 hours. Then, the cells were harvested, cultured, and expanded according to reported methods^30^. The chondrocytes in passage 2 were harvested for the construction of tubular cartilage (Fig. 2A).

### Engineering of cell-scaffold constructs

The harvested chondrocytes were resuspended in DMEM containing 10% fetal bovine serum (FBS; Hyclone. Logan, UT, USA) to a final concentration of 5.0 *10^7^ cells/ml. After soaking the scaffold in cultured medium to moisten it, the scaffold was again soaked with a 2 ml cell suspension in a 15 ml centrifugal tube with 5% CO_2_ at 37 °C for 30 minutes and was reversed every ten minutes to ensure equal cell adherence on all sides of the scaffold. Then the scaffold was transfered gently into 6 well cell culture clusters (Becton Dickinson, NJ, USA) with 7.5 ml DMEM containing 10% FBS, 50 μg/ml of vitamin C (Sigma Aldrich, MO, USA), 100 U/mL of penicillin, 100 mg/mL of streptomycin, and 0.1 mM nonessential amino acids (all from Invitrogen Co. Carlsbad, CA, USA). After 3 days culture, all clusters were put on a shaking table at 100 rpm with 5% CO_2_ and 37 °C for 2 or 4 weeks.

### Observation by scanning electron microscopy (SEM)

To detect the cytocompatibility of the scaffold, the blank scaffold, 2-weeks cultured TET and 4-weeks cultured TET was observed by SEM in Shanghai Jiao Tong University, Bio-X Institutes. All samples were fixed in 4% paraformaldehyde for 24 h and dehydrated with graded concentrations (50%, 70%, 80%, 90%, 95% and 100% v/v) of ethanol. Subsequently, the samples were coated with gold/palladium and observed by SEM Quanta 250 (FEI, USA).

### Subcutaneous implantation in nude mouse

Four nude mice (7-weeks old) were distributed into two experimental groups: Group I, TET cultured *in vitro* for 2 weeks or Group II, TET cultured *in vitro* for 4 weeks. Each sample was implanted into the dorsal subcutaneous spaces of each nude mouse. Animal care and housing was provided by Experimental Animals Department of Shanghai Children’s Medical Center. After 6 weeks, all implants were retrieved from each mouse for macrograph and histological analysis.

### Replacement of native trachea in rabbits and postoperative care

20 rabbits were divided randomly into two groups: Group I, TET cultured *in vitro* for 2 weeks (n = 8), group II, TET cultured *in vitro* for 4 weeks (n = 12). All rabbits were anesthetized with intravenous injection of pentobarbital sodium (30 mg/kg). Under sterile conditions, a midline incision was made in the anterior neck. Surrounding tissue was separated carefully to expose the trachea. An approximate 1.6 cm segment of trachea was cut off at 1.0 cm below the cricoid cartilage. A tissue-engineering trachea was placed into the defect trachea and performed end-to-end continuous anastomosis with 6–0 absorbable PDS II sutures (Johnson & Johnson, NJ, USA) in both proximal and distal native trachea. Then incision was closed in layers and the animal was allowed to breathe spontaneously and recover from anesthesia.

Postoperatively, all rabbits were given intramuscular injection of cefuroxime for 5 days to prevent infection. Animal care and monitoring was provided by Experimental Animals Department of Shanghai Children’s Medical Center. Once the animals died, the survival period was recorded, autopsy was performed to analyze the causes of death. The implanted trachea including part of native trachea was dissected from surrounding tissues and taken out. To observe the stenosis of the inner lumen and granulation formation, the implanted trachea was cut off ring by ring for gross view and histology analysis.

### Histological analysis

All histology staining were performed as previously described^30^. The samples were fixed in 4% paraformaldehyde, embedded in paraffin, and then sectioned into 8 μm sections. The sections were stained with hematoxylin and eosin (H&E) to assess tissue structure and Toluidine blue and Safranin O to locate the Glycosi Aminoglyca Good deposits.

### Statistical analysis

All the quantitative data were recorded as mean ± standard deviation. SPSS 23.0 statistical software (SPSS Inc., Chicago, IL, USA) was used for statistical analysis. Survival rates were estimated with the Kanlan-Meier method and Breslow test. A p-value less than 0.05 were considered as statistically significant.
